# Ingroup Bias in Healthcare Contexts: Israeli-Jewish Perceptions of Arab and Jewish Doctors

**DOI:** 10.3389/fpsyg.2021.771028

**Published:** 2021-12-16

**Authors:** Elliot Graham, Samer Halabi, Arie Nadler

**Affiliations:** ^1^Department of Behavioral Sciences, Academic College of Tel-Aviv-Yaffo, Yaffo, Israel; ^2^Department of Psychological sciences, Tel Aviv University, Tel Aviv, Israel

**Keywords:** ethnic bias, group membership, ingroup bias, physician ethnicity, ingroup favoritism, physician bias

## Abstract

The influence of group membership on perceptions of outgroup members has been extensively studied in various contexts. This research has indicated a strong tendency for ingroup bias – preferring the ingroup over the outgroup. We seek to further expand on the growing literature regarding the effects of group membership within healthcare contexts. Focusing on the Arab-Jewish context in Israel, the present study explored the influence of group membership on Israeli-Jewish participants’ evaluations when exposed to potential malpractice. Specifically, participants (*n* = 165) read a description of an Israeli-Jewish or Israeli-Arab physician who was either culpable or non-culpable of malpractice. Consistent with our predictions, findings generally indicated more negative evaluations of the Israeli-Arab physician, regardless of objective culpability. We conclude by discussing the study’s limitations and implications.

## Introduction

We live in a globalized world where we are continuously exposed to other groups, ethnicities, and cultures. Examining the effects of individuals’ membership in groups that interact daily has therefore become increasingly relevant. Indeed, extensive research has been conducted to explore the important effects of group membership in intergroup relations across multiple life spheres (Allport, 1954; Tajfel, 1978; Pettigrew and Tropp, 2008). Israel, 20% of whose citizens belong to the Arab minority, provides an ideal context for studying how group membership can impact intergroup relations between that minority and the Jewish majority. In what follows, we focus on a specific and understudied setting: healthcare. Specifically, we investigated how a physician’s group membership may influence Israeli-Jews’ evaluations of that physician following potential malpractice.

Israel’s public health system is a microcosm of its diverse social environment. In particular, there has recently been significant growth in the number of Israeli-Arabs employed in the medical and paramedical professions. Israeli-Arabs represent 19.2% of medical students and 14.7% of physicians (Averbuch and Avni, 2018; Israel Central Bureau of Statistics, 2018). Daily intergroup contact – such as Israeli-Jewish patients treated by Israeli-Arab physicians and *vice-versa* – may influence patients’ perceptions of the specific medical processes, as well as general attitudes toward the provider and their group. Indeed, several qualitative studies conducted within the Israeli health system have reported on the way conflicted relations between Arabs and Jews in Israel affect attitudes toward outgroup caregivers, such as the preference for physician–patient concordance (or ingroup matchup; Popper-Giveon and Keshet, 2018; Popper-Giveon, 2019). In fact, an example of the conflicted relations within a medical setting could be observed when a member of the Israeli parliament was reported to have backed Jewish women who requested to be separated from Arab women while staying in maternity wards, a request that some hospitals were reported to have agreed to Siegel-Itzkovich and Solomon (2016). The present research focuses primarily on understanding the influence of the above relationship by exploring the effects of a physician’s group membership on the perceptions of Israeli-Jewish participants regarding the extent of a physician’s hypothetical culpability following potential malpractice.

Physicians usually enjoy high social status in addition to being the most respected profession among both groups of Israeli citizens (Haruti-Sover, 2017; Kadan et al., 2019). As such, it would be important to explore how Jewish members of this high-status group react to an Arab physician compared to Jewish physician. Indeed, research has indicated that members of high-status groups may recognize and inhibit obvious forms of discrimination, and yet be more affected by implicit negative stereotypes that are often remain subconscious (Stone and Moskowitz, 2011). Consequently, they may attribute more blame to outgroup than to ingroup members (Halabi et al., 2015). Pursuing this line of research in healthcare system, specifically in Israel, is most important as group membership is most salient and may trigger negative social perceptions that may affect judgments, evaluations, and behavior of potential patients.

Throughout life, individuals associate themselves and others with those who share a similar social identity – a social grouping defined as an *ingroup* – while those who differ are considered *outgroup* members (*we* and *them*; Tajfel, 1978; Abrams and Hogg, 1998). This recognition of others as in- or outgroup members has a robust, general effect on the perceptions and evaluations of others by displaying a preference for ingroup over outgroup members – or *ingroup bias* (Turner et al., 1979; Tajfel and Turner, 1986; Brown, 2019). Consequently, people tend to process information more deeply about their ingroup as opposed to any outgroup (Van Bavel et al., 2008). Additionally, people generally recall more unfavorable information about outgroup than about ingroup members (Howard and Rothbart, 1980). Furthermore, individuals evaluate ingroup members more positively and allocate resources more generously to ingroup than to outgroup members (Guimond, 2000; see Dovidio and Gaertner, 2010, for a review), especially when the intergroup context is salient, as postulated by social identity theory (Tajfel and Turner, 1986).

Importantly, people tend to attribute culpability for adverse outcomes differently for in- and outgroup members. For example, a study on linguistic intergroup bias (Maass et al., 1989) revealed a tendency to describe positive ingroup and negative outgroup behaviors in abstract terms, while describing negative ingroup and positive outgroup behaviors in concrete terms. For example, undesirable actions by outgroup members were perceived in a way that presumed intentionality and dispositional origin more than identical behaviors of ingroup members. Additional research showed that people more strongly attribute positive behaviors and successful outcomes to internal, stable characteristics of ingroup than outgroup members, whereas they ascribe the causes of adverse outcomes more strongly to the personalities of outgroup than ingroup members (Pettigrew, 1979; Hewstone, 1990).

In the current study, we extend these findings and explore the influence of group membership while focusing on the Israeli healthcare context. Specifically, we investigate the interethnic effects that may influence the evaluations of Israeli-Jewish participants regarding possible malpractice by an Israeli-Jewish vs. Israeli-Arab physician. Extensive research has indicated to the complicated relationships between Arabs and Jews in Israel (e.g., Enos and Gidron, 2016; Suleiman et al., 2018). Due to both historical, cultural, and contemporary political factors, the relationship between Israeli-Jews and Israeli-Arabs has been one of antagonism and conflict, creating a climate of suspicion, mistrust, and threat that has posed real difficulties to the engagement in shared living (Bar-Tal et al., 2010). Furthermore, these pervasive tensions between the two groups have contributed to the preservation and reinforcement of the ongoing discrimination and exclusion of Arabs in Israel (Smooha, 2014). At the same time, signs of rapprochement can be seen as well, as observed in the reduction in radicalization and improved attitudes between the groups observed from 2017 to 2019 (Smooha, 2020).

Studies conducted on the effects of intergroup relations in Israel have found that certain cues serve to categorize others as outgroup members, increasing biases in the process. One such study found that while positive imagined interaction with Arab-Muslim group members reduced intergroup anxiety among Jewish participants, the effect was significantly weakened when the Arab-Muslim member was perceived to display identifying Muslim religious symbols (Razpurker-Apfeld and Shamoa-Nir, 2020a). Another study conducted by Razpurker-Apfeld and Shamoa-Nir (2020b) presented similar results in regard with regard to motor cues, with arm extension found to increase intergroup anxiety and prejudice. These findings are consistent with those of a similar study on the effects of aural cues such as accents on enhancing ingroup biases (Jiang et al., 2020).

This tension may manifest itself within the Israeli healthcare system, where Israeli-Arabs and Jews interact daily as patients and healthcare providers. Indeed, the influence of the complicated relations between Jews and Arabs in Israel on the Israeli medical healthcare system has been observed in a studies that explored both physician–patient and physician–nurse interactions (Popper-Giveon et al., 2014; Keshet and Popper-Giveon, 2017; Popper-Giveon and Keshet, 2018). For example, Popper-Giveon et al. (2014) found that while Israeli-Arab physicians supportive of integration did not necessarily display a preference for patient concordance, both Israeli-Arab and Jewish patients would often rather be treated by physicians from their own ethnic group (see also Popper-Giveon, 2019). Further studies conducted in medical clinics in Israel found that positive intergroup contact could help reduce intergroup prejudice and improve attitudes both in a natural setting and in those based on positive information (Weiss, 2020, 2021). In light of these studies, we must also consider the positive effects found of patient-physician concordance on general health outcomes, as well as the likelihood of survival and correct treatment (Alsan et al., 2019; Greenwood et al., 2020).

### The Present Study

In the current study, we focused on Jews, the majority group in Israel, and explored their response to a hypothetical case in which a physician, presented as an Israeli-Jew or Israeli-Arab, was responsible for malpractice that caused severe damage to a patient. Based on a previous study that indicated the influence of group membership on perceptions regarding culpability (Maass et al., 1989), we focused on variables that would represent the effects of group membership on the physician-patient relationship following a negative outcome. In particular, we assessed participants’ emotions toward the physician, their trust in the physician, and the severity of punishment that they believed the physician deserved. We predicted that participants would display more negative attitudes toward an Arab compared to a Jewish physician across all our dependent variables and, importantly, regardless of the physician’s culpability.

Considering the negative influence of group membership on perceived culpability when blame is not clear, a situation where blame can be more readily attributed should present an even “clearer” context; in which more negative reactions are made toward the culpable outgroup members (Maass et al., 1989; Schruijer et al., 1994). This is particularly so where contact is brief and impersonal, as in a hospital emergency room, with brief contact found to increase the effect of in-group biases (Forbes, 2004; Füchtbauer et al., 2013). Therefore, we expected to find a significant effect for Physician’s Group Membership (Israeli-Jew vs. Israeli-Arab) × Culpability (High vs. Low) interactions regarding emotions expressed toward the physician, degree of trust in the physician, and the severity of punishment considered appropriate for the physician. Further, based on previous research that focused on the role of group-based emotions play in fueling and maintaining intergroup conflicts (Halperin, 2016) and on the role of trust in establishing positive intergroup relations (Bar-Tal et al., 2017), we also tested whether more positive emotions and more trust in the Israeli-Arab physician would mediate attributing, similar to the Israeli-Jewish physician, less severe punishment for the Israeli-Arab physician even when the physician is perceived as responsible for a potential malpractice.

## Materials and Methods

### Participants and Design

Following approval from the institutional review board, a total of 163 Israeli-Jews (102 women) were recruited to participate in the study on voluntarily basis. Participants were recruited through advertisements in various social media platforms, using a convenience sampling method. We published a call for participants in a study involving real-life incidents to examine laypersons’ information processing regarding the world of medicine. A link was provided within the post directing candidates to our questionnaire, which was conducted on Qualtrics. Participants then filled out an informed consent form with our contact information for any further inquiries. Data of all participants were retained and used for the analysis resulting in zero exclusion.

Ranging in age from 18 to 72 (*M* = 28.16; *SD* = 7.82), the participants were randomly assigned to one of four conditions within a 2 (Phynosician’s Group Membership: Israeli-Jewish vs. Israeli-Arab) × 2 (Culpability: High vs. Low) between-subjects design. The goal was to recruit as many participants as possible up to an *n* of 128 (based on *a priori* statistical power analysis using G*Power version 3.1; Faul et al., 2009) to test all direct, interactive, and simple effects within a 2 × 2 factorial design with 80% power and a medium effect size. *Post-hoc* sensitivity analysis (Faul et al., 2009) showed a small effect size, *f* = 0.25 (Cohen, 1988). Thus, we had sufficient power to test our key predictions.

### Procedure

The participants were informed they were participating in a study on how people perceived and reacted to real-life incidents involving social interactions. Following a general description of the study, they were asked to read a paragraph from an article published in a well-known newspaper. The paragraph supposedly included a description of a real-life event involving a physician and patient. Then, following the procedure employed by Halabi et al. (2017), the paragraph described a male physician called Alon, a common and exclusively Israeli-Jewish name, for the first group of participants (*n* = 68). For the other group of participants (*n* = 95), the male physician described in the paragraph was named Mahmoud, a common and exclusively Arab name^1^. Following this group membership manipulation, the participants were asked to state the physician’s name and mother tongue. These items were designed to test the effectiveness of our group membership manipulation.

Next, the physician’s culpability was manipulated. Specifically, participants were asked to read a brief paragraph including a description of a “real-life incident” that occurred in an Israeli hospital. In this incident, Yossi, a Jewish patient experienced irreversible damage following a severe allergic reaction to a presurgical sedative. Then, for approximately half of the participants (*n* = 83), the paragraph described the Jewish patient as being under stress and, when questioned by the physician, as failing to report any medicinal allergies, representing the low-culpability condition. In contrast, in the high-culpability condition, participants (*n* = 80) were presented with a case where the on-call physician was under stress in the emergency room and consequently did not inquire about any patient allergies.^2^

To examine the effectiveness of our culpability manipulation, we asked the participants to rate their agreement on whether the physician was culpable in the incident on a five-point scale ranging from 1 (*highly*) to 5 (*not at all*). Specifically, participants were asked, “To what extent is the physician culpable in the case you just read?”

Next, participants were asked to indicate their feelings and reactions regarding what they had just read. The following were our dependent variables: (a) emotions toward the physician, (b) trust in the physician, and (c) the severity of punishment that participants thought the physician deserved.

#### Emotions Toward the Physician

Emotions toward the physician have been linked to patients’ positive affective states and a greater satisfaction with their physician (Hall et al., 2002; Worthington and Scherer, 2004). Alternatively, it was found that a lesser degree of outwardly expressed positive emotion by patients could lead to more negative caretaker dispositions (Ogrodniczuk et al., 2008). Participants were asked to rate on a five-point scale their emotions, toward the physician using four items adapted from Ogrodniczuk et al. (2008) ^3^: (1) “To what degree do you feel sympathy toward the physician involved?”; (2) “To what degree do you feel admiration toward the physician involved?”; (3) “To what degree would you be willing to forgive the physician involved?”; and (4) “To what degree do you feel anger toward the physician involved?” (reverse-coded; Cronbach’s alpha = 0.72). The items were averaged, with higher scores reflecting more positive emotions toward the physician.

#### Trust in the Physician

Trust has been found to play a significant role in positive patient-physician relationships in addition to being linked to patient satisfaction and adherence to treatment (Pearson and Raeke, 2000; Hall et al., 2001). Trust in the context of the patient-physician relationship is a multilayered construct that includes the patient’s belief in a physician’s competence and their confidence that their best interest is the primary consideration (Becker, 1996). Using a five-point scale, participants were asked to rate scale their trust in the physician *via* four items: (1) “To what degree would you trust this physician if he were treating you?”; (2) “To what degree would you recommend this physician to your friends?”; (3) “Compared to other physicians, would you positively evaluate the physician involved?”; and (4) “To what degree would you be willing for this physician to be your own?” (Cronbach’s alpha = 0.88). The items were averaged, with higher scores reflecting higher trust.

#### Severity of Punishment

Based on previous studies that investigated the relationship between group membership and recommended severity of punishment (Halabi et al., 2015), participants were asked to rate on a five-point scale the severity of the punishment they thought the physician deserved *via* three items: (1) “Do you believe that the physician’s license should be revoked?”; (2) “Do you believe that the physician should be criminally charged?”; and (3) “If you were the patient involved, would you have sued the physician?” (Cronbach’s alpha = 0.75). The items were averaged, with higher scores reflecting a more severe punishment.

## Results

Preliminary analysis showed no significant effects for participants’ gender; thus, this factor was not considered in the subsequent analysis. We then tested the effect of perceived culpability and the physician’s group membership manipulations on the participants’ emotions toward the physician, the extent of their trust in the physician, and the severity of punishment they believed that the physician deserved.

### Manipulation Checks

To test the effectiveness of the physician’s perceived culpability manipulation, a 2 (Physician’s Group membership: Israeli-Jew vs. Israeli-Arab) × 2 (Culpability: High vs. Low) ANOVA was conducted on participants’ level of agreement with the statement that the physician was culpable for the incident. This analysis revealed only the predicted main effect for the culpability manipulation, *F*(1,164) = 41.25, *p* < 0.001, *η*^2^ = 0.160. Supportive of the intended manipulation, participants in the high-culpability condition (*M* = 1.95, *SD* = 0.953) perceived the physician as significantly more responsible than in the low-culpability condition (*M* = 3.02, *SD* = 1.33). No significant effects were found for physician group membership (*p* = 0.873) or for the interaction effect (*p* = 0.090).

For the group membership manipulation, all the participants correctly identified the physician’s name and mother tongue according to the experimental condition.

### Ratings of the Physician

With respect to our main outcomes of interest, we conducted a 2 (Physician’s Group membership: Israeli-Jew vs. Israeli-Arab) × 2 (Culpability: High vs. Low) between-subjects ANOVAs separately for indexes of emotions toward the physician, trust in the physician, and severity of punishment. Means and SDs of dependent variables across the study conditions are presented in Table 1.

**Table 1:** Means (SDs) of emotions towards the Physician, Trust in the Physician, and Severity of Punishment as a Function of Physician’s Ethnicity and Culpability.

Physician’s Ethnicity					Experimental condition	Israeli-Jewish		Israeli-Arab	
**Culpability:**		High (*n* = 32)	Low (*n* = 36)	High (n = 48)	Low (*n* = 47)
**Dependent measures:**				
Emotions towards the Physician	3.01 (0.82)	3.07 (0.91)	2.59 (0.78)	2.81 (0.84)
Trust in the Physician	2.69 (0.89)	2.49 (1.06)	2.29 (0.79)	2.78 (0.89)
Severity of Punishment	2.63 (0.97)	2.74 (1.14)	3.30 (0.87)	2.67 (0.80)

**Figure 1:**
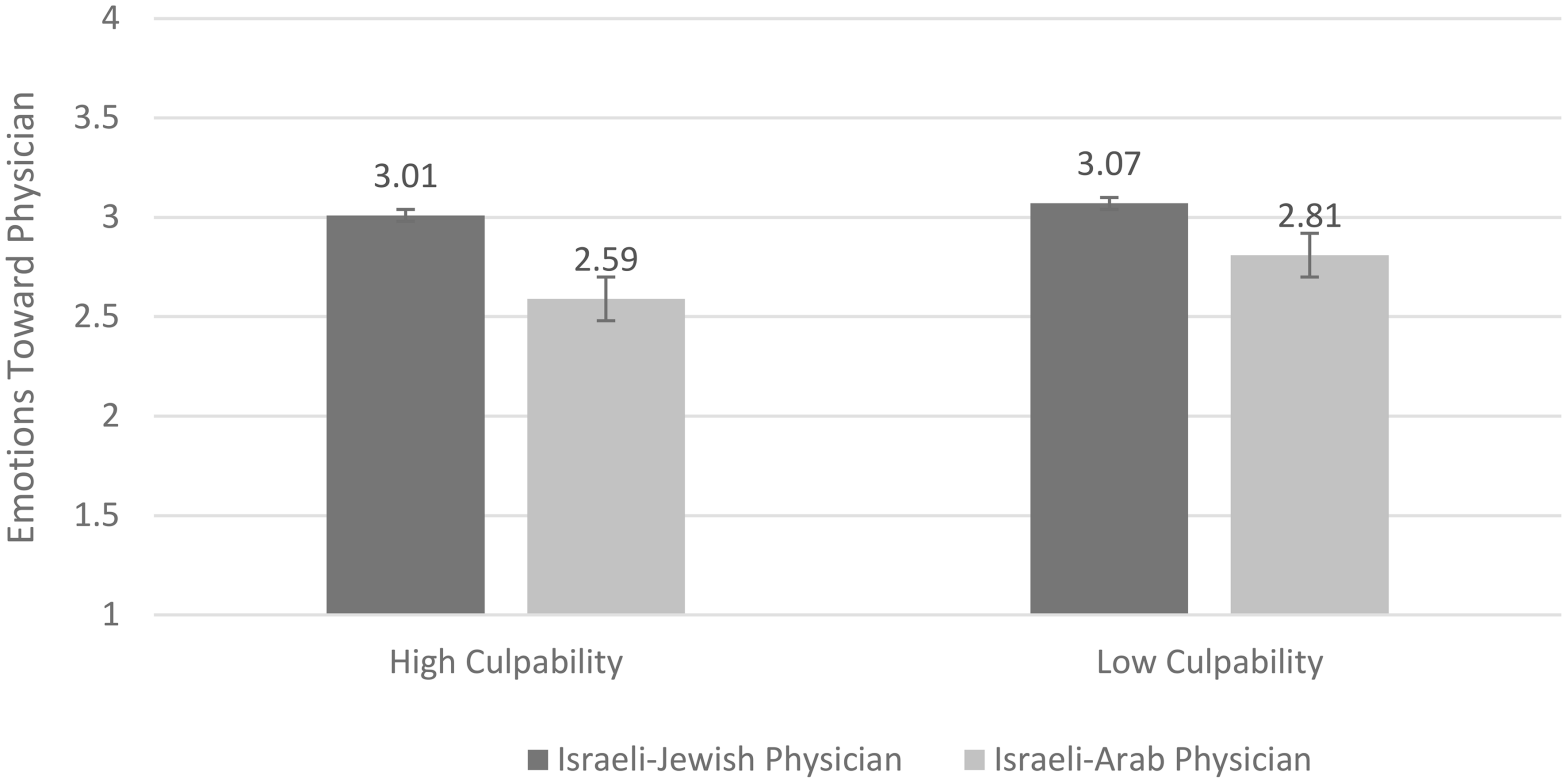
Emotions toward Physician as a Function of Physician’s Ethnicity and Culpability.

For *emotions toward the physician*, the ANOVA demonstrated a significant main effect for ethnicity, *F*(1,162) = 6.29, *p* = 0.01, *η*^2^ = 0.04. Participants reported more positive emotions toward the Israeli-Jewish physician than the Israeli-Arab physician, *M*s = 3.04 (*SD* = 0.86) vs. 2.7 (*SD* = 0.81). The main effect of culpability was not significant, *F*(1,162) = 1.15, *p* = 0.28, *η*^2^ = 0.01. Additionally, the Physician’s group membership × Culpability (see Figure 1) interaction was not significant, *F*(1,162) = 0.25, *p* = 0.61, *η*^2^ = 0.002.

**Figure 2:**
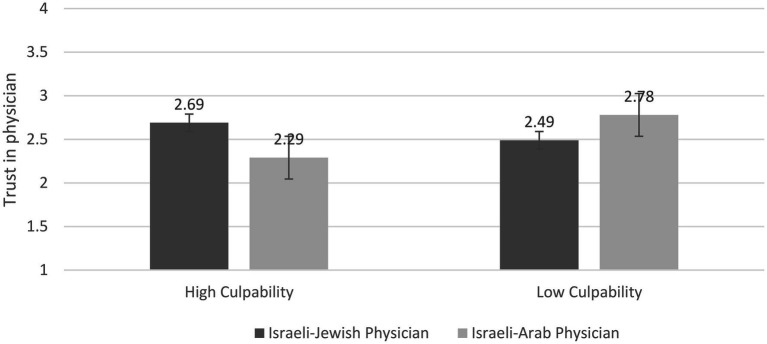
Trust in Physician as a Function of Physician’s Ethnicity and Culpability.

A 2 (Physician’s Group Membership) × 2 (Culpability) ANOVA on *trust in the physician* revealed only the significant two-way interaction, *F*(1,162) = 5.66, *p* = 0.019, *η*^2^ = 0.034. Further analysis showed that participants in the high-culpability condition reported slightly higher trust in the Israeli-Jewish physician than in the Arab physician, *M*s = 2.69 (*SD* = 0.89) vs. 2.29 (*SD* = 0.79), respectively, *t*(159) = 1.92, *p* = 0.06, *d* = 0.47. However, the comparable difference in the low-culpability condition did not approach significance, *M*s = 2.49 (*SD* = 1.08) vs. 2.78 (*SD* = 0.89), respectively; *t*(159) = 1.43, *p* = 0.15, *d* = 0.96. Looked at differently, *post hoc* analysis showed that in the Israeli-Arab physician condition, participants reported significantly higher trust in the physician in the low-culpability than in the high-culpability condition, *M*s = 2.78 (*SD* = 0.89) vs. 2.29 (*SD* = 0.79), respectively; *t*(159) = 2.60, *p* = 0.01, *d* = 0.57. However, in the Israeli-Jewish physician condition, the degree of trust did not differ between the high- and low-culpability conditions, *M*s = 2.69 (*SD* = 0.89) vs. 2.49 (*SD* = 1.06), respectively; *t*(159) = 0.92, *p* = 0.36, *d* = 0.22 (see Figure 2).

**Figure 3:**
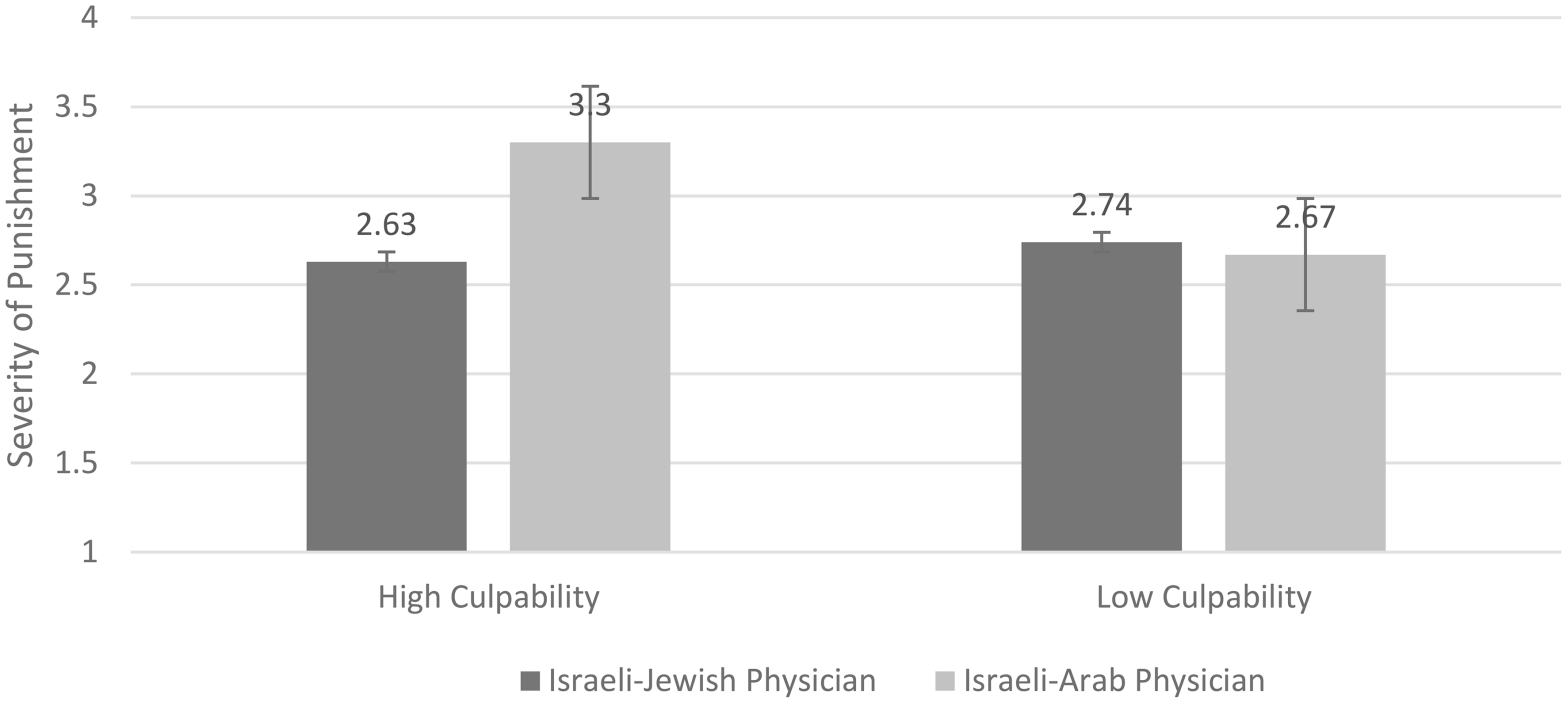
Severity of Punishment as a function of Physician’s Ethnicity and Culpability.

Finally, a 2 (Physician’s Group Membership) × 2 (Culpability) ANOVA on *severity of punishment* revealed a significant main effect for ethnicity, *F*(1,162) = 4.07, *p* = 0.04, *η*^2^ = 0.025. As expected, participants attributed more severe punishment to the Israeli-Arab physician than to the Israeli-Jewish physician, *Ms* = 2.99 (*SD* = 0.89) vs. 2.69 (*SD* = 1.05), *d* = 0.31. Furthermore, as predicted, a significant two-way Group Membership of Physician × Culpability interaction effect was found, *F*(1,162) = 6.07, *p* = 0.01, *η*^2^ = 0.04. Further analysis showed that participants in the high-culpability condition attributed significantly more severe punishment to the Israeli-Arab than to the Israeli-Jewish physician, *M*s = 3.30 (*SD* = 0.877) vs. 2.63 (*SD* = 0.970), respectively; *t*(159) = 3.12, *p* = 0.002, *d* = 0.72. However, in the low-culpability condition, no such significant difference was found, *M*s = 2.67 (*SD* = 0.8) vs. 2.74 (*SD* = 1.14), respectively; *t*(159) = 0.33, *p* = 0.75, *d* = 0.1. Interestingly, when observing the participants’ perceptions regarding the Israeli-Jewish physician, no statistically significant differences were found between the high- and low-culpability conditions in severity of punishment, *Ms* = 2.63 (*SD* = 0.97) vs. 2.74 (*SD* = 1.14), respectively; *t*(159) = 0.46, *p* = 0.64, *d* = 0.1. Furthermore, note that, no statistical difference was found between the high-culpability-Israeli-Jewish physician and low-culpability-Israeli-Arab physician conditions, *Ms* = 2.63 (*SD* = 0.970) vs. 2.67 (*SD* = 0.8), respectively; *t*(159) = 0.39, *p* = 0.69, *d* = 0.04 (see Figure 3).

## Discussion

The present study examined the effects of group membership on perceptions of Israeli Jews in the understudied context of the healthcare system. Specifically, we explored Israeli-Jewish evaluations of male physicians presented as either Israeli-Arab or Israeli-Jewish. Our findings illuminate the crucial role that a physician’s ethnicity may play in eliciting positive or negative emotions of ingroup vs. outgroup members toward them, as well as perceptions regarding the severity of punishment that the physician deserved following potential malpractice. Furthermore, the current findings presented that participants were more likely to be influenced by the Israeli-Arab physician’s group membership in ratings of trust in the physician when the physician was presented as more responsible for the malpractice. Specifically, Israeli-Jewish participants reported more negative emotions toward the Israeli-Arab physician than toward the Israeli-Jewish physician. Furthermore, when the physician was presented as more responsible for potential malpractice the participants reported a higher degree of trust and attributed the less severe punishment toward the Israeli-Jewish physician when compared to the Israeli-Arab physician. Importantly, the severity of punishment attributed to the physician in the high-culpability condition was mediated by emotions reported toward the physician and by extent of trust that participants displayed toward him.

Our results show how objective culpability and ethnicity intertwine, with participants significantly more likely to display more negative emotions toward Israeli-Arab physicians and assign them more punishment than Israeli-Jewish ones, when presented as culpable for a medical malpractice. The current findings indicate the harmful effects that group membership can have on an individual’s emotions toward an outgroup physician. Additionally, when considering our findings on the influence of group membership on the severity of punishment, the significance of the above becomes clearer, further emphasizing how the needs of punishment and positive emotions such as forgiveness can often be intertwined (Worthington et al., 2005).

As predicted, the influence of group membership on the desire for punishment and the capacity for trust was significantly more evident in cases where the Israeli-Arab physician’s culpability was high, while no such effect was found where the culpability of the physician (of whatever ethnicity) was low. These results emphasize the impact that culpability can have in exposing and enhancing ingroup bias – specifically, the harmful role that an Israeli-Arab physician’s culpability plays in Israeli-Jewish group members’ trust and desire for punishment. When questioning how these influences may impact an Israeli-Arab physician, we can begin by reiterating the impact that a patient’s trust in a physician has on the quality of their interaction (Pearson and Raeke, 2000; Hall et al., 2001). We also might speculate how this trust could potentially display itself regarding an Israeli-Arab’s employer once an unfortunate incident occurs. Additionally, we can consider how the observed group bias impacts Israeli-Jews’ perceptions regarding the severity of punishment that Israeli-Arab and -Jewish physicians deserve following medical malpractice, potentially affecting a vast number of legal cases.

Looked at differently, the current findings provide an interesting perspective in understanding prejudice and stereotyping when outgroup members of higher social status are involved. In particular, Israeli-Jewish participants reacted more negatively to Israeli-Arab physicians only when they were to blame for medical malpractice. This suggests that when the outgroup member, even with high social status, behaves in a way that “reminds” the ingroup observer of the other’s status affiliation, interpersonal perceptions are likely to be affected by this affiliation more than by the higher status (Halabi and Nadler, 2021). However, this important theoretical extension needs to be further examined in future research.

Crucially, one of our most significant findings was the lack of observable difference, across all variables, between the high- and low-culpability Israeli-Jewish physicians. Additionally, we found no observable differences between the low-culpability Israeli-Arab physician and high-culpability Israeli-Jewish physician. This lack of observed differences between the Israeli-Jewish physicians could be problematic in terms of their ability to learn from their errors, affecting the system as a whole. Conversely, the results regarding the lack of observable differences between the low-culpability Israeli-Arab physician and the high culpability Israeli-Jewish physician, in conjunction with the significant difference between the Israeli-Arab physicians across all variables, suggest that Israeli-Jews are more critical of Israeli-Arab physicians, expecting less, and once a mistake is made, retroactively assuming that they have been generally less professional. These results strongly emphasize, in a medical context, that group membership can influence perceptions, such as people’s capacity to judge situations objectively and perceive culpability when members of their ingroup are involved (Hewstone, 1990; Holmberg and Kyvsgaard, 2003).

Several possible explanations may be provided for the uneven results in several of our variables. A number of our participants may have felt weaker identification with their ingroup, thus influencing their sense of group homogeneity, a substantial ingredient in reducing the negative influence of group membership (Wilder, 1978; Dietz-Uhler and Murrell, 1998; Hutchison et al., 2006). Future studies could benefit from expanding upon the possible moderating influence of group identification on group membership bias within a medical setting. Additionally, the results showed no significant difference between the Israeli-Jewish and Israeli-Arab physicians in the low-culpability condition. It is conceivable that, by inquiring into the nature of the patient’s allergies, the Israeli-Arab physician appeared to be showing more concern, thus reducing the effects of intergroup bias (Giannakakis and Fritsche, 2010).

## Limitations and Future Directions

The first limitation of this study is that whereas its theoretical implications present an opportunity to understand the relationship between Israeli-Jews and their Israeli-Arab physicians, due to the absence of Israeli-Arab participants, we are unable to conclude that all types of ingroup membership lead to discrimination against outgroup physicians. Future studies should attempt to build an additional sample consisting of Israeli-Arabs.

Additionally, the nature of this study, conducted on a general population sample, prevents us from observing the nature of group membership bias within an actual emergency care medical environment, such as the one presented in our study. While studies such as this allow for greater degree of control, prior research conducted regarding ingroup bias toward Arabs in Israel indicated multiple benefits for studies conducted using natural experiment settings, such as naturally occurring groups, professional judgement, and higher stakes (Shayo and Zussman, 2011; Grossman et al., 2016; Bar and Zussman, 2020). Further research may greatly benefit from a similar study, with a more gender-balanced sample, held in a hospital setting with a patient-focused sample, as well with hospital administrations that may be also affected by ethnicity and perceived physician culpability. Such a study may represent the population more appropriately and strengthen the validity of our theoretical and applied findings. Furthermore, while our results yielded a statistically significant difference regarding observable discrimination towards Israeli-Arab physicians, seeing as our results generally clustered around the midway point we must consider that this difference may not always be as perceivable in real life scenarios.

Finally, our study only attempted to observe how group membership could create a bias in perceptions of the physician-patient relationship. For that reason, we believe that future studies conducted in the field should attempt to find specific methods to reduce the impact of group membership bias on the physician-patient relationship. For example, considering the positive effects found for physician-patient concordance within other medical settings, examining the impact of concordance within an emergency room using a natural experiment may help underscore the need for increasing physician diversity. Other studies may focus on how moderating factors such as spreading positive information in the media may help reduce bias within a framework such as that presented here (Ortiz and Harwood, 2007; Weiss, 2020).

## Conclusion

Using an experimental design, we built upon previous evidence for the existence of group membership bias in the healthcare context. Our study persuasively presents the existence of such bias against Israel-Arab physicians within the Israeli health system, implying that this effect can probably be found within actual physician-patient relationships, especially if the physician is suspected of malpractice.

These harmful influences – resulting from ethnic ingroup bias – could have grave real-world consequences, and as observed in our study, they can influence emotions toward the physician, trust in the physician, and the severity of punishment in cases of suspected malpractice. These could dramatically affect an Israeli-Arab physician’s career path and the quality of physician–patient interaction (Pearson and Raeke, 2000; Baicker et al., 2007; Shurtz, 2013). This impact is crucial considering the current rise in the number of Israeli-Arab physicians employed throughout the Israeli healthcare system.

We believe that the path to reducing the harmful influences of group membership begins with their deeper understanding. Hopefully, the information provided in this study serves as a significant step in the healthcare context.

## Data Availability Statement

The raw data supporting the conclusions of this article will be made available by the authors, without undue reservation.

## Ethics Statement

The studies involving human participants were reviewed and approved by Institutional Review Board. The patients/participants provided their written informed consent to participate in this study.

## Author Contributions

EG was responsible on data collection and data analysis and writing research report. SH was responsible on designing the study, and involved in data analysis and writing. AN was involved in supervising and writing research report. All authors contributed to the article and approved the submitted version.

## Funding

This research was partially supported by a research grant from the Israeli Science Foundation (grant no. 1288/16) awarded to SH and AN.

## Conflict of Interest

The authors declare that the research was conducted in the absence of any commercial or financial relationships that could be construed as a potential conflict of interest.

## Publisher’s Note

All claims expressed in this article are solely those of the authors and do not necessarily represent those of their affiliated organizations, or those of the publisher, the editors and the reviewers. Any product that may be evaluated in this article, or claim that may be made by its manufacturer, is not guaranteed or endorsed by the publisher.

## Footnotes

^1^It should be noted that the two conditions of physician group membership vary in size, 68 participants in the Israeli-Jewish condition vs. 95 participants in the Israeli-Arab condition. However, we believe that this did not affect our randomization process, as the experiment was conducted *via* the Qualtrics online platform. Indeed, as in all sound experimental studies, the random assignment of participants to the different conditions is the hallmark of a “true experiment.” While random assignment can be accomplished by complete randomization, in which half of participants are randomly assigned to the control and half to the experimental condition, it can also be achieved by simple randomization, in which for each participant that volunteers for the experiment, there is a 50/50 chance that she/he ends up in the control or in the experimental condition – regardless of how large either sample already is. Simple randomization, as administered in the current study, may have caused the unequal sample sizes, yet it may have notably reduced the potential risk for selection bias (see Kahan et al., 2015) as the experimental software takes care of the randomization process.

^2^This culpability manipulation was piloted prior to the main study. Forty Israeli-Jewish participants were randomly recruited *via* Facebook to test the consistency and validity of our manipulation. As in the main study, participants were presented with a “news report.” For approximately half of them, the physician could be perceived as more culpable (*n* = 22), while the other half received a report where the physician’s culpability was low (*n* = 20). The results demonstrated that the manipulation was correctly understood (*M =* 2.37, *SD = 0*.92 versus *M =* 1.57, *SD = 0*.56; *t*(30) = 3.32, *p* = 0.017, *d* = 1.05).

^3^It should be mentioned that the original measure used by Ogrodniczuk et al. (2008) was not a self-reported scale and focused on caretakers’ reactions toward the patient, thus differing from our study which may have influenced results. Furthermore, the original measure used broad terms for positive and negative emotions. In the current study, the emotions scale, adapted from Ogrodniczuk et al. (2008) focused on assessing emotions that previous studies on reactions to receiving help found to be indicative in describing the emotional state of participants.
