# Supplementary material for: Ingroup Bias in Healthcare Contexts: Israeli-Jewish Perceptions of Arab and Jewish Doctors
Source: Front Psychol. 2021 Dec 16;12:771028. doi: 10.3389/fpsyg.2021.771028 (PMC8716498; doi:10.3389/fpsyg.2021.771028)
Supplement: Supplementary file 1 [file data_sheet_1.docx]

**Appendix**

*Arab Physician – High Culpability:*

יוסי, שכבר מספר ימים מרגיש לא טוב, חווה החמרה במצבו ומחליט ללכת לחדר מיון. בחדר המיון, הרופא התורן מחמוד, קובע כי הוא סובל מדלקת חריפה בתוספתן וכי הוא זקוק לניתוח בהול. עקב העומס במטופלים בחדר המיון, ולאור הדחיפות בניתוח, **ד"ר מחמוד** מורה לאחות להכין את המטופל לניתוח, אך מיד נקרא בדחיפות למקרה חירום לפני שהוא מספיק לבדוק את ההיסטוריה הרפואית של המטופל כמתחייב מהכללים הרפואיים, בה מצוין במפורש כי הוא סובל מרגישות נדירה לחומר ההרדמה.

במהלך הניתוח הבחין **ד"ר מחמוד** כי חום גופו של המטופל זינק באופן דרסטי והגיע לכ 42 מעלות. בנוסף, נוצרה אי סדירות מטרידה בקצב לבו. לאחר מאמצים רבים, שנמשכו מספר שעות בהם היה שרוי במצב מסכן חיים, הצליח הצוות הרפואי להוריד את חום גופו. לאחר התייצבותו, וחזרתו להכרה, התגלה כי האירוע שהתרחש במהלך הניתוח גרם לנזק בלתי הפיך שהותירה אותו מרותק לכיסא גלגלים.

Yossi, who had been feeling unwell for several days, experiences a worsening of his condition and decides to go to the emergency room. In the emergency room, the doctor on duty, Mahmoud, states that he suffers from acute inflammation in their appendix and that he needs urgent surgery. Due to an overcrowding of patients in the emergency room, and considering the urgency of the surgery, Dr. Mahmoud instructs the nurse to prepare the patient for surgery but is immediately called away for an emergency, this happened before examining the patient's medical history as required by medical protocol.

During the operation, Dr. Mahmoud noticed that the patient's body temperature had increased drastically and reached about 42 degrees. In addition, there was a disturbing irregularity in the patient’s heart rate. With considerable effort, which lasted several hours in which he was in a life-threatening condition, the medical staff was able to lower his body temperature. Once the patient had stabilized, and regained consciousness, it was discovered that the incident that occurred during the surgery caused irreversible damage which subsequently left him confined to a wheelchair.

*Arab Physician- Low Culpability:*

יוסי, שכבר מספר ימים מרגיש לא טוב, חווה החמרה במצבו ומחליט ללכת לחדר מיון. בחדר המיון, הרופא התורן  ד"ר **מחמוד**, קובע כי הוא סובל מדלקת חריפה בתוספתן וכי הוא זקוק לניתוח בהול. יוסי, מתוך הכאבים בהם היה שרוי והלחץ מדחיפות הניתוח, שכח לציין בפני **ד"ר מחמוד** כי יש לו רגישות נדירה לחומר ההרדמה.

במהלך הניתוח הבחין**ד"ר מחמוד** כי חום גופו של יוסי זינק באופן דרסטי והגיע לכ42 מעלות. בנוסף, נוצרה אי סדירות מטרידה בקצב לבו. לאחר מאמצים רבים, שנמשכו מספר שעות בהם היה שרוי במצב מסכן חיים, הצליח הצוות הרפואי להוריד את חום גופו. לאחר התייצבותו, וחזרתו להכרה, התגלה כי האירוע שהתרחש במהלך הניתוח גרם לנזק בלתי הפיך שהותירה אותו מרותק לכיסא גלגלים.

Yossi, who had been feeling unwell for several days, experiences a worsening of his condition and decides to go to the emergency room. In the emergency room, the doctor on duty, Mahmoud, states that he suffers from acute inflammation in their appendix and that he needs urgent surgery. Yossi due to the pain he was in as well as the stress brought on by the urgency of the surgery, forgot to point out to Dr. Mahmoud that he has a rare sensitivity to the anesthetic.

During the operation, Dr. Mahmoud noticed that the patient's body temperature had increased drastically and reached about 42 degrees. In addition, there was a disturbing irregularity in the patient’s heart rate. With considerable effort, which lasted several hours in which he was in a life-threatening condition, the medical staff was able to lower his body temperature. Once the patient had stabilized, and regained consciousness, it was discovered that the incident that occurred during the surgery caused irreversible damage which subsequently left him confined to a wheelchair.

*Jewish Physician- High Culpability:*

יוסי, שכבר מספר ימים מרגיש לא טוב, חווה החמרה במצבו ומחליט ללכת לחדר מיון. בחדר המיון, הרופא התורן מחמוד, קובע כי הוא סובל מדלקת חריפה בתוספתן וכי הוא זקוק לניתוח בהול. עקב העומס במטופלים בחדר המיון, ולאור הדחיפות בניתוח, **ד"ר אלון** מורה לאחות להכין את המטופל לניתוח, אך מיד נקרא בדחיפות למקרה חירום לפני שהוא מספיק לבדוק את ההיסטוריה הרפואית של המטופל כמתחייב מהכללים הרפואיים, בה מצוין במפורש כי הוא סובל מרגישות נדירה לחומר ההרדמה.

במהלך הניתוח הבחין **ד"ר אלון** כי חום גופו של המטופל זינק באופן דרסטי והגיע לכ 42 מעלות. בנוסף, נוצרה אי סדירות מטרידה בקצב לבו. לאחר מאמצים רבים, שנמשכו מספר שעות בהם היה שרוי במצב מסכן חיים, הצליח הצוות הרפואי להוריד את חום גופו. לאחר התייצבותו, וחזרתו להכרה, התגלה כי האירוע שהתרחש במהלך הניתוח גרם לנזק בלתי הפיך שהותירה אותו מרותק לכיסא גלגלים.

Yossi, who had been feeling unwell for several days, experiences a worsening of his condition and decides to go to the emergency room. In the emergency room, the doctor on duty, Alon, states that he suffers from acute inflammation in their appendix and that he needs urgent surgery. Due to an overcrowding of patients in the emergency room, and considering the urgency of the surgery, Dr. Alon instructs the nurse to prepare the patient for surgery but is immediately called away for an emergency, this happened before examining the patient's medical history as required by medical protocol.

During the operation, Dr. Alon noticed that the patient's body temperature had increased drastically and reached about 42 degrees. In addition, there was a disturbing irregularity in the patient’s heart rate. With considerable effort, which lasted several hours in which he was in a life-threatening condition, the medical staff was able to lower his body temperature. Once the patient had stabilized, and regained consciousness, it was discovered that the incident that occurred during the surgery caused irreversible damage which subsequently left him confined to a wheelchair.

*Jewish Physician- Low Culpability:*

יוסי, שכבר מספר ימים מרגיש לא טוב, חווה החמרה במצבו ומחליט ללכת לחדר מיון. בחדר המיון, הרופא התורן  ד"ר **אלון**, קובע כי הוא סובל מדלקת חריפה בתוספתן וכי הוא זקוק לניתוח בהול. יוסי, מתוך הכאבים בהם היה שרוי והלחץ מדחיפות הניתוח, שכח לציין בפני **ד"ר אלון** כי יש לו רגישות נדירה לחומר ההרדמה.

במהלך הניתוח הבחין**ד"ר אלון** כי חום גופו של יוסי זינק באופן דרסטי והגיע לכ42 מעלות. בנוסף, נוצרה אי סדירות מטרידה בקצב לבו. לאחר מאמצים רבים, שנמשכו מספר שעות בהם היה שרוי במצב מסכן חיים, הצליח הצוות הרפואי להוריד את חום גופו. לאחר התייצבותו, וחזרתו להכרה, התגלה כי האירוע שהתרחש במהלך הניתוח גרם לנזק בלתי הפיך שהותירה אותו מרותק לכיסא גלגלים.

Yossi, who had been feeling unwell for several days, experiences a worsening of his condition and decides to go to the emergency room. In the emergency room, the doctor on duty, Alon, states that he suffers from acute inflammation in their appendix and that he needs urgent surgery. Yossi due to the pain he was in as well as the stress brought on by the urgency of the surgery, forgot to point out to Dr. Alon that he has a rare sensitivity to the anesthetic.

During the operation, Dr. Alon noticed that the patient's body temperature had increased drastically and reached about 42 degrees. In addition, there was a disturbing irregularity in the patient’s heart rate. With considerable effort, which lasted several hours in which he was in a life-threatening condition, the medical staff was able to lower his body temperature. Once the patient had stabilized, and regained consciousness, it was discovered that the incident that occurred during the surgery caused irreversible damage which subsequently left him confined to a wheelchair.
