# Supplementary material for: Ingroup Bias in Healthcare Contexts: Israeli-Jewish Perceptions of Arab and Jewish Doctors
Source: Front Psychol. 2021 Dec 16;12:771028. doi: 10.3389/fpsyg.2021.771028 (PMC8716498; doi:10.3389/fpsyg.2021.771028)
Supplement: Supplementary file 2 [file data_sheet_2.docx]

**Supplementary Analyses**

Although it was not the main purpose of the current study, we also tested wether the effects of the manipulations on severity of punishment are mediated by emotions and trust in the physician. Using the PROCESS macro (Hayes, 2012; Model 8 with 10000 bootstrap resamples), we found, as expected, that group membership of physician in the high culpability condition, but not in the low culpability condition, had a significant effect on emotions toward the physician, and emotions towards the physician had a significant effect on severity of punishment, b = .271, SE = .126, 95%CI [.052, .538]. We also tested for the hypothesized mediated moderation effect – the effect of the Physician’s Group Membership x Culpability interaction on severity of punishment, mediated by trust in the physician. The results revealed that, as expected, there was a significant indirect path from the Physician’s Group Membership x Culpability interaction towards severity of punishment through trust in the physician, b = -.397, SE = .186, 95%CI [-.790,- .064]. The indirect effect of Physician’s Group Membership had a significant positive effect on trust towards the physician, and trust in the physicians had a significant effect on severity of punishment, in the high culpability condition, b = .231, SE = .179, 95%CI [.0206, .490]. In the low culpability condition, this indirect effect was non-significant, b = -.167, SE = .131, 95%CI [-.436, .103].

Culpability (High/ Low)

Emotions/Trust toward physician

Severity of punishment

Physician’s group membership

Figure 1**.** Theoretical moderated mediation model based on Hayes' (2012) process model 8, with Physician’s Group Membership (Jew vs. Arab) as independent variable, Culpability (high vs. low) as moderator, emotions toward the physician and trust in physician as mediators, severity of punishment as dependent variable.
